# Analysis of the Characteristics and Cytotoxicity of Titanium Dioxide Nanomaterials Following Simulated In Vitro Digestion

**DOI:** 10.3390/nano10081516

**Published:** 2020-08-02

**Authors:** Ana Bettencourt, Lídia M. Gonçalves, Ana C. Gramacho, Adriana Vieira, Dora Rolo, Carla Martins, Ricardo Assunção, Paula Alvito, Maria João Silva, Henriqueta Louro

**Affiliations:** 1 Research Institute for Medicines (iMed.ULisboa), Faculty of Pharmacy, Universidade de Lisboa, 1649-003 Lisboa, Portugal; asimao@ff.ulisboa.pt (A.B.); lgoncalves@ff.ulisboa.pt (L.M.G.); 2 Department of Human Genetics, National Institute of Health Dr. Ricardo Jorge (INSA), Avenida Padre Cruz, 1649-016 Lisboa, Portugal; ana.gramacho@insa.min-saude.pt (A.C.G.); adriana.vieira@insa.min-saude.pt (A.V.); dora.rolo@insa.min-saude.pt (D.R.); M.Joao.Silva@insa.min-saude.pt (M.J.S.); 3 Food and Nutrition Department, National Institute of Health Dr. Ricardo Jorge (INSA), Avenida Padre Cruz, 1649-016 Lisboa, Portugal; carla.martins@insa.min-saude.pt (C.M.); ricardo.assuncao@insa.min-saude.pt (R.A.); Paula.Alvito@insa.min-saude.pt (P.A.); 4 CESAM-Centre for Environmental and Marine Studies, University of Aveiro, Campus Universitário de Santiago, 3810-193 Aveiro, Portugal; 5 NOVA National School of Public Health, Public Health Research Centre, Universidade NOVA de Lisboa, Avenida Padre Cruz, 1600-560 Lisboa, Portugal; 6 ToxOmics–Centre for Toxicogenomics and Human Health, NOVA Medical School, Universidade NOVA de Lisboa, Campo dos Mártires da Pátria, 130, 1169-056 Lisboa, Portugal

**Keywords:** nanomaterials, ingested titanium dioxide, standardized in vitro digestion, cytotoxicity

## Abstract

Several metallic nanomaterials (NMs), such as titanium dioxide nanomaterials (TiO_2_), present beneficial properties with a broad range of innovative applications. The human population is exposed to TiO_2_, particularly by ingestion, due to its increasing use as a food additive and inclusion in dietary supplements and food packaging materials. Whether this oral exposure may lead to adverse local or systemic outcomes has been the subject of research, but studies have generated contradictory results, reflecting differences in the physicochemical properties of the TiO_2_ studied, effects of the surrounding matrix, and modifications during digestion. This work aimed to investigate the toxic effects of three different TiO_2_ NMs (NM-103, NM-103 and NM-105) on the gastrointestinal tract cells, Caco-2 and HT29-MTX-E12, after the use of the standardized static INFOGEST 2.0 in vitro digestion method to mimic human digestion of TiO_2_, contributing to hazard assessment. The results show that, for one of the digested TiO_2_ NMs studied (NM-105), a more pronounced toxicity occurs after exposure of HT29-MTX-E12 intestinal cells, as compared to undigested NM, concomitantly with subtle changes in characteristics of the NM. Thus, the inclusion of the digestion simulation in the safety evaluation of ingested NMs through in vitro bioassays can better integrate the modifications that NMs suffer in the organism. It is expected that such an approach will reduce uncertainties in the hazard assessment of ingested NMs for human health.

## 1. Introduction

The technology based on manufactured nanomaterials (NMs) has been highlighted as a key enabling technology, due to its potential to improve many products and processes, namely in the agriculture, food and feed industry [1]. Many of such products available have metallic NMs, silver or titanium dioxide nanomaterials (TiO_2_) and many others are being developed, such as nanocellulose and nanoformulations of pesticides [1,2,3]. The oral exposure may occur either directly, through the consumption of products/pharmaceuticals or foods containing NMs, or indirectly, through the ingestion of foods contaminated with NMs released from food-contact materials (packaging, refrigerator coatings, storage containers or other equipment and coatings) or from environmental sources [4]. Therefore, ingestion appears to be a probable route of exposure to NMs, and the gastrointestinal tract (GIT) may be the first site of contact of the ingested NMs allowing a systemic exposure if the intestinal barrier is surpassed [5,6]. Accordingly, human volunteer studies support the systemic uptake following ingestion of TiO_2_ particles [7,8]. Furthermore, the internal exposure to TiO_2_ has been demonstrated, since TiO_2_ nanoparticles have been detected in post-mortem samples from human liver and spleen most likely accumulated through ingestion [9].

Until now, there is no consensus on the use of TiO_2_ as a food additive. The European Food Safety Authority (EFSA) Panel on “Food Additives and Nutrient Sources Added to Food” stated that the food additive E171 containing TiO_2_ (anatase or rutile structure) does not raise a genotoxic concern [10,11]. However, other regulatory bodies such as the French Agency for Food, Environmental and Occupational Health and Safety (ANSES) have emphasized the need to conduct studies to fully characterize the potential health effects related to ingestion of this food additive [12], leading to the ban of its use in food in France, since January 2020. 

The major concern is that TiO_2_ may produce adverse outcomes such as genotoxic effects that are associated with increased risk of cancer. Although NMs have been extensively investigated in recent years, contradictory results have been generated, possibly due to differences in the physicochemical properties of the NMs studied and to other variables in the test systems. In fact, we previously showed that NMs with the same chemistry, but differing in primary properties, e.g., TiO_2_ varying in size, surface area and crystalline phase, or multiwalled carbon nanotubes differing in length, diameter, the aspect ratio or degree of flexibility, may yield different biological effects [13,14]. Furthermore, the NMs properties have been recognized as being context-dependent, i.e., can be affected by the surrounding matrix. For example, the agglomeration status of metallic NMs dispersion used for cells exposure seems to influence the observed genotoxic effects [15]. Likewise, the authors reported that these secondary features might be potentially more relevant than the primary properties for determining toxicological outcomes. Concerning ingested NMs, it is predictable that processes like human digestion may modify the NMs characteristics leading to unexpected toxicity in intestine cells. Since GIT is chemically and physically complex, ingested NMs pass through different pH and chemical environments prior to their intestinal uptake, which may affect their physicochemical properties such as size, surface charge and morphology.

The general approaches to evaluate the potential toxicity of chemicals, also applied to NMs, generally involve a battery of in vitro and in vivo tests. Concerning in vivo assays, it has been suggested that the impact on the gastrointestinal tract should be re-evaluated because of significant differences in the physiology and nutrient uptake of the GIT between humans and rats, hampering interspecies extrapolation [16]. Conversely, most in vitro assays using human cells do not account for the potential modifications of the NMs under physiological conditions and processes, e.g., following digestion. NMs may agglomerate, react or bind with other components of food/feed, solubilize on reaction with stomach acid or digestive enzymes, or be excreted from the body [17]. Due to such transformations, NMs may not be available in free particulate forms, which influences its translocation across the GIT and thus cytotoxic outcome [18].

To simulate the digestion of food, several in vitro models have been used for many decades. However, the diversity of methods available include important variations of digestion parameters such as pH, duration, enzyme concentration and activity and composition of simulated digestive fluids, which hampers the comparison between laboratories and studies. The international INFOGEST network included multidisciplinary experts (in, e.g., food science, nutrition, gastroenterology, engineering and enzymology) from more than 35 countries for developing, for the first time, an international consensus on a set of digestion parameters for a static in vitro simulation of adult digestion suitable for food. The method, generally referred to as the INFOGEST method, was published [19] and experimental parameters were justified and discussed in great detail in relation to available in vivo physiological data. An interlaboratory trial applying different in vitro digestion protocols clearly demonstrated the good reproducibility of the standardized INFOGEST protocol [20]. An amended and improved digestion method (INFOGEST 2.0) avoiding challenges associated with the original method, such as the inclusion of the oral phase and the use of gastric lipase, was then published [21] and disseminated online [22]. Recently, the standardized INFOGEST 2.0 in vitro digestion method has been shown to be a valuable tool for addressing the in vitro digestion of food contaminants [23] and eventually NMs [18] thereby providing an alternative to animal models.

In spite of many papers reporting the cytotoxicity assessment of TiO_2_ in cells from GIT (see for example [24,25]), none has addressed and compared the effects upon digestion process of different crystalline TiO_2_ forms in two distinct cell lines. A recent work studied the dissolution behavior, biodurability and persistence of several NMs (TiO_2_, SiO_2_, ZnO and Fe_2_O_3_) in individual simulated gastrointestinal fluids (saliva, gastric and intestinal) and a physiologically relevant digestion cascade (saliva–gastric–intestinal), showing that TiO_2_ was the most biodurable and persistent NM [26]. However, in the later no results of biological effects were reported. Using a fasting dietary model, the presence of E171 can result in cytotoxic effects [27]. In addition, the work by Deloid et al. recently studied the impact of gastrointestinal effects on the biokinetics and cellular toxicity of ingested engineered nanomaterials, but it was applied only to iron oxide NMs [18].

Here, we report the use of the validated standardized INFOGEST 2.0 in vitro digestion method [21] to deliver a more realistic approach for studies on the cytotoxicity of TiO_2_ on the GIT, and as a tool to further contribute to its hazard assessment. Our general working hypothesis is that digestion processing of the TiO_2_ with different primary characteristics will result in different secondary properties that further influence their toxicological impact. The objectives of this study were two-fold: i) to characterize the physicochemical properties of a set of three distinct TiO_2_ after the simulated digestion process, comparing them with the primary NMs’ properties; ii) to explore the impact of the digestion process on the NMs’ cytotoxicity in two human-derived intestinal cell lines. Overall, the results revealed that the digested NMs had no major cytotoxicity in both cell lines, except for one of the TiO_2_ studied, NM-105, where a more pronounced adverse outcome was observed in HT29-MTX-E12 intestinal cells after digestion, as compared to undigested NM (not subjected to simulated digestion process). NM-105 also showed a lower size of the agglomerates/aggregates upon digestion process that may explain its increased toxicity.

## 2. Materials and Methods 

### 2.1. Nanomaterials Properties and Dispersion for Biological Assays

The TiO_2_ were provided by the Joint Research Centre (JRC, Ispra, Italy) and included NM-102, NM-103 and NM-105. All the NMs were prepared under good laboratory practices (GLP), allowing their application as international benchmarks. Their physicochemical characteristics have been previously described and are summarized in Table 1 [28].

These three TiO_2_ have distinct crystalline structures and different hydrodynamic sizes, specific surface area and agglomerate size. In addition, NM-103 is hydrophobic, Al-coated, while others are uncoated. NM-105 (also known as Aeroxide P25) has been selected as principal material for the Organisation for Economic Co-operation and Development (OECD) test program “Testing a representative set of manufactured nanomaterials” [28]. It exhibits mixed crystallinity, with anatase as the predominant form (81.5% anatase:18.5% rutile).

A 2.56 mg/mL stock dispersion of each NM was prepared by prewetting powder in 0.5% absolute ethanol (96%) followed by addition of sterile-filtered 0.05 wt % BSA-water and dispersion by 16 min of probe sonication of the sample, cooled in an ice-water bath [29]. The stock dispersions were immediately used either for the static digestion process (obtaining the digested samples) or directly (corresponding to the undigested samples) for physicochemical characterization and biological assays, after dilution in cell culture medium, as described in Section 2.4.

### 2.2. Simulation of In Vitro Digestion

This standardized method was based on an international consensus on a set of digestion parameters for a static in vitro simulation of adult digestion suitable for food developed by the COST INFOGEST network. The digestion protocol used followed the standardized INFOGEST 2.0 in vitro digestion method suitable for food, as previously described [21]. The samples were digested in the upper GIT, food particles are broken down in the mouth, stomach and small intestine through complex reactions and interactions involving chemical and mechanical processes. Briefly, the method is detailed below.

#### 2.2.1. Chemicals and Reagents

The following reagents were used to prepare the simulated digestion fluids: CaCl_2_(H_2_O), KCl, NaHCO_3_, NaCl, MgCl_2_(H_2_O)_6_, NaOH (Merck, Darmstadt, Germany), (NH4)_2_CO_3_ (Sigma-Aldrich, St. Louis, MO, USA), KH_2_PO_4_ and HCl (J. T. Baker, Center Valley, PA, USA). The enzymes α-Amylase, pepsin, bile, pancreatin and Pefabloc^®^ SC were purchased from Sigma-Aldrich^®^ (St. Louis, MO, USA).

#### 2.2.2. In Vitro Digestion Procedure

The present protocol considered three sequential phases: oral, gastric and intestinal. The simulated fluids, i.e., simulated salivary fluid (SSF; pH 7), simulated gastric fluid (SGF; pH 3) and simulated intestinal fluid (SIF; pH 7) were prepared in accordance with reference [21]. Briefly, for the oral phase, 1 mL of each NM were mixed with 1 mL of SSF with α-amylase (75 U/mL, pH 7). Concerning the gastric phase, 2 mL of SGF with pepsin (2000 U/mL, pH 3) were added and for the intestinal phase, 4 mL of SIF with pancreatin (100 U/mL of trypsin activity, pH 7) and bile (10 mM, pH 7) were added. The incubation of the digestion tubes at 37 °C were performed using a mechanical shaker for two minutes (oral phase) and 120 min (gastric and intestinal phases). After intestinal phase incubation, the enzymatic reactions were stopped with addition of 5 mM of Pefabloc^®^ (Sigma-Aldrich, St. Louis, MO, USA). The in vitro digestion protocol considered that enzymes must be incorporated according to their enzyme activity [21]. Enzyme activity and bile salt concentration assays were performed according to the methodologies detailed [21].

The digestion end products (digested samples) were used to assess the effect of in vitro digestion on NMs’ properties as well as on their cytotoxicity.

### 2.3. Effect of the In Vitro Simulated Digestion on the NMs Properties 

The effect of in vitro simulated digestion on NMs properties was assessed by three different techniques: 1) dynamic light scattering (DLS); 2) electrophoretic light scattering and 3) transmission electron microscopy (TEM) coupled to X-ray energy dispersive spectrometry (EDS). The dispersions were analyzed 10–30 min after sonication and dilution in complete cell culture medium with 15% fetal bovine serum, as described in Section 2.4.

DLS was used to determine the hydrodynamic particle size-distribution and the polydispersity index of the NMs dispersions as described previously [14]. DLS measurements were conducted on a Zetasizer Nanoseries Nano S (Malvern Instruments, Malvern, UK) using automatic optimization of analytical conditions and data treatment by a general purpose size-analysis. The hydrodynamic size (Zav, nm) and polydispersity index (PDI) for each NM was expressed as the mean of 3 consecutive measurements.

Electrophoretic light scattering using a Zetasizer Nanoseries Nano Z (Malvern Instruments, Malvern, UK) measured the zeta potential of the NMs dispersions. All measurements were performed in triplicate and the results are shown as the mean ± standard deviation (SD).

TEM analysis conducted on a Hitachi H-8100 transmission electron microscope (Tokyo, Japan) operating at 200 kV enabled to assess the morphology and the size of the primary and secondary (agglomerates/aggregates) of the NMs. TEM observation was assessed by dropping a suspension of the NMs batch dispersion or digestion product onto a formvar-coated copper grid that was subsequently air-dried. The elemental chemical composition was determined by the respective EDS, using a light elements detector from ThermoNoran and the software Noran System (SIX. ThermoNoran, Madison, WI, USA).

The iTEM software (Hitachi, Tokyo, Japan) was used for the quantitative TEM analysis of the primary NMs and agglomerates/aggregates using the arbitrary line tool. For each sample, 10 micrographs were taken at a magnification of 40,000 times. The maximum Feret diameter corresponding to the length of the particle (Feret Max) and the minimum Feret diameter (Feret Min) were measured. The Feret mean of the particle was calculated as the mean of Feret Min and Feret Max. The aspect ratio was calculated as the ratio of Feret Max and Feret Min. The size range estimation of the agglomerates/agglomerates/aggregates was obtained considering the maximum and minimum dimension of each in the micrographs [30].

### 2.4. Cell Culture and Cytotoxicity Assays

Two human intestinal cell lines (Caco-2 and HT29-MTX-E12 cells), obtained from the European Collection of Authenticated Cell Cultures (ECACC), were selected as experimental models for in vitro studies. The human colon adenocarcinoma cell line Caco-2 was chosen due to its source tissue (human colon), the characteristics resembling human enterocytes and its widespread use in in vitro studies. The human colorectal adenocarcinoma cells HT29-MTX-E12 were selected as an alternative intestinal model, with the ability of producing a mucous layer, allowing ascertaining the influence of the mucous layer on nanoparticle diffusion.

Caco-2 and HT29-MTX-E12 cells were grown in Dulbecco’s modified Eagle medium (DMEM, Thermo Fisher, Waltham, MA, USA) supplemented with 1% penicillin/streptomycin (10,000 U/mL), 2.5% HEPES buffer, 10% or 15% fetal bovine serum (FBS, for HT29-MTX-E12 or Caco-2, respectively) and 1% fungizone (all from Thermo Fisher). Cells were maintained at 37 °C in a humidified atmosphere with 5% CO_2_.

Preliminary data indicated a high cytotoxicity of the digestion product itself, i.e., without NM. The solution of BSA-water used to disperse the NMs (DIG0) was submitted to the digestion process and the final product was added to the cell culture medium at various dilutions. We observed that dilutions of that digestion product above 12.5% in the cell culture medium (equivalent to final NMs concentration higher than 28 μg/mL) were cytotoxic (data not shown). Considering also previous reports [30,31], predicting that 0.14 μg/mL is a realistic level of human intestinal epithelium exposure following oral intake, we selected the concentrations of 0, 0.14, 1.43 and 14.3 μg/mL of NM to perform the cytotoxicity assay.

For the MTT assay, Caco-2 cells and HT29-MTX-E12 were cultured at a cell density of 1 × 10^4^ cells/well in 96-well plates and incubated for 24 h, 37 °C, 5% CO_2_ before the exposure. Cells were exposed to the selected concentrations of the undigested or digested TiO_2_, after dilution in the cell culture medium. The negative controls included BSA-water (vehicle) and digestion product (without NM) exposed cells. Cells were exposed to sodium dodecyl sulphate (SDS, 0.01%), used as positive control, for 1 h. At the exposure time, the cell medium was replaced with the treatment medium and incubated for 24 h at 37 °C, 5% CO_2_. Three independent replicates were performed per exposure condition. After 24 h of exposure, the cell medium was removed, cells were washed with PBS and 3-(4,5-dimethylthiazol-2-yl)-2,5-diphenyltetrazolium bromide (MTT, Sigma-Aldrich) solution (0.5 mg/mL) was added to each well. Cells were incubated for an additional 3 h. The MTT solution was removed and DMSO was added to each well and incubated for 30 min. The absorbance of each well was measured in a Multiskan Ascent Spectrophotometer (Thermo LabSystems, Waltham, MA, USA) at 570 nm (reference filter: 690 nm). The viability (%) of the treated cells was defined as the percentage of absorbance compared to control untreated cells (100% viability). A reduction in viability corresponds to an increased cytotoxicity.

### 2.5. Statistical Analysis 

The statistical analyses of the results were performed using Prism software (6.01, GraphPad, San Diego, CA, USA) and SPSS Statistics (20, IBM, Armonk, NY, USA). A Mann–Whitney U test was applied to TEM data. A one-way ANOVA and post-hoc tests were applied to MTT data of each undigested and digested NM. To compare the various treatment conditions, a Student’s *t*-test was applied. Values of *p < 0.05* was considered statistically significant.

## 3. Results and Discussion

In the present study, different techniques such as DLS, zeta potential and TEM-EDS were used for evaluating the effect of simulated GIT media on NMs primary and secondary physicochemical properties as next described, before using the digested NMs for cytotoxicity assays.

### 3.1. Hydrodynamic Size Distribution and Surface Charge

The hydrodynamic size (Zav, nm), polydispersity index (PDI) and surface charge (zeta potential, mV) for each NM were obtained shortly after the dilution of batch dispersions in the cell culture medium and are presented in Table 2. While NM-102 showed a narrow distribution with a peak in 459 nm, NM-103 and NM-105 had a primary peak in the range of 190–220 nm. Furthermore, NM-103 also showed a secondary peak at ca. 1106 nm, suggestive of multimodal distribution, as previously reported [14]. In general, size-distributions could be seen with no major impact of digestion on Zav and small NMs with less than 100 nm were still detectable in dispersion.

The size distribution of the undigested samples is within the range of previously reported for these NMs [13]. Compared to the size-range of DLS reported by the manufacturer in the batch dispersion (Table 1), higher Zav was obtained for all NMs diluted in cell culture medium. This is consistent with previous reports showing that the presence of serum proteins resulted in NM agglomeration and corona formation, a phenomenon known in nanotoxicology research [32]. Nevertheless, the cellular assays hereby described are only feasible using serum-containing medium to allow normal cell division among other functions, and we consider that they mimic real-life conditions since under physiological conditions, such a phenomenon will also occur.

The DLS data demonstrated, with respect to undigested samples, that a monodisperse dispersion was achieved only for NM-102, which presented the lowest PDI values. NM-103 presented a Zav with a high standard deviation, due to multimodal distribution and the presence of a secondary peak, as previously mentioned. NM-105 presented a PDI value greater than 0.7 indicating that the sample has a broad size distribution due to the presence of agglomerates. However, upon digestion, this NM shows a decrease in PDI, suggesting a better dispersion, and the mean size is significantly lower than the one from undigested sample (*p* = 0.0042, Student’s *t*-test). Therefore, NM-105 appears to agglomerate less upon digestion, while the other NMs agglomerate more after digestion process.

Concerning a surface charge, both undigested and digested NMs presented negative zeta potentials, with similar values between −20 and −30 mV (Table 2), being an indicator of moderately stable dispersion systems [33]. Negative zeta potentials, lower than −30 mV, were previously reported for these NMs [28]. Similar values were obtained after digestion, in accordance to other simulated digestion models, where strong negative charges have been reported, with and without iron NMs, that became more moderate upon dilution in the cell culture medium [18]. The results show that charge was a physicochemical parameter not affected by the digestion process reflecting no dissimilarities in the amount, composition or structure of the adsorbed components of the digestion media (oral, gastric or intestinal) around each type of NM [18]. This data is in accordance with others who showed that inorganic TiO_2_ NMs had little impact on the gastrointestinal fate of the lipids of simulated gastrointestinal fluids suggesting stable electrostatic interactions between the inorganic NMs and the organic components of the digestion media [34].

### 3.2. TEM-EDS Analysis

**Figure 1:**
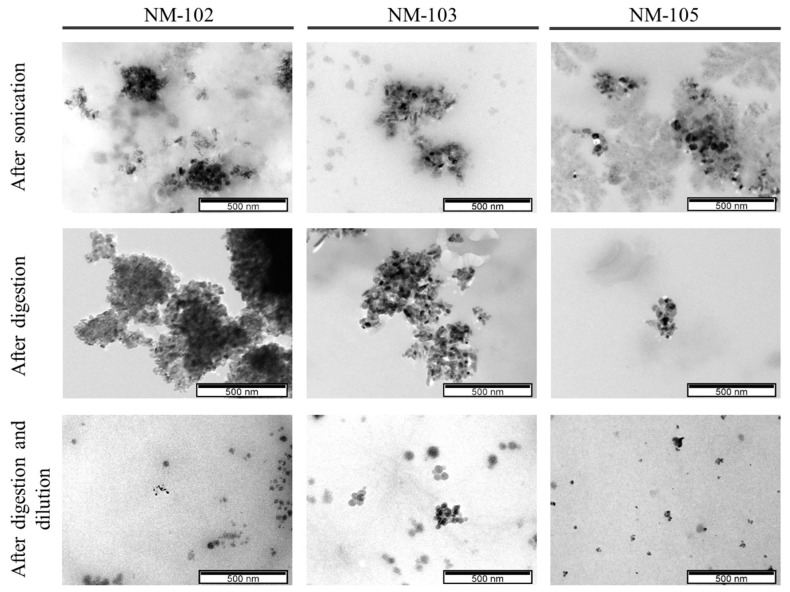
TiO_2_ observed under electron microscopy, at a scale of 500 nm.

**Figure 2:**
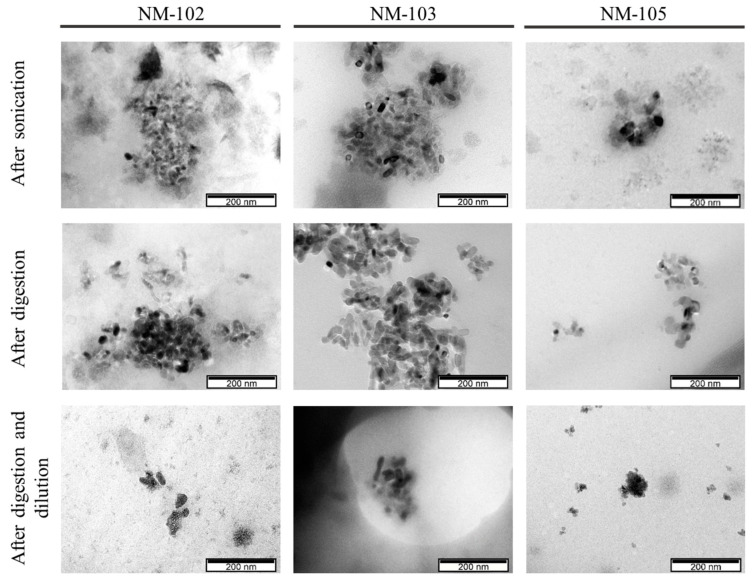
TiO_2_ observed under electron microscopy, at a scale of 200 nm.

**Figure 3:**
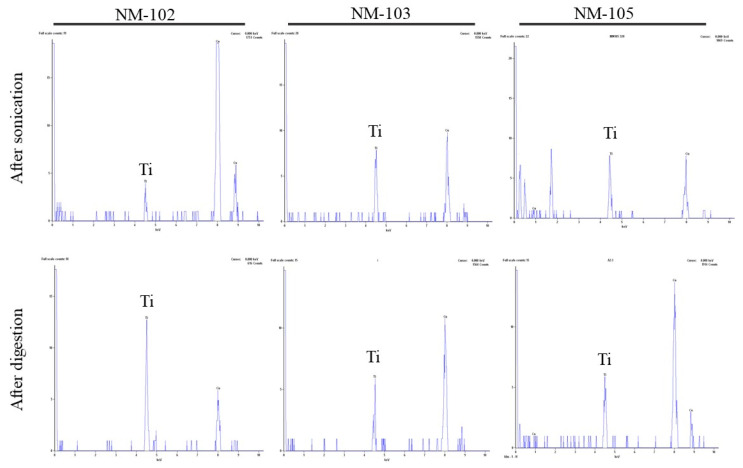
Representative elemental analysis spectra by TEM-EDS. Note: Ti corresponds to titanium and Cu identifies the copper element present in TEM grid.

Representative micrographs of the NMs before and after digestion are presented in Figure 1 and Figure 2, while the presence of elemental titanium was confirmed by EDS (Figure 3).

TEM images were used to obtain size measurements but also specific shape properties, distinguishing between characterization of primary particles and agglomerates/aggregates. 

From the multiple images obtained (only representative images are shown), all the NMs, with or without digestion, consist of single particles < 100 nm and agglomerates/aggregates as already demonstrated in the DLS experiments. The general morphology of the primary units of the NMs is elongated and rounded, suggesting an ellipsoidal structure, consistent with previous reports from the NMs’ provider [28]. The agglomerates/aggregates tend to have a more fractal-like structure. The mean size and aspect ratio that were determined in TEM images are presented in Table 3.

The primary size (Feret min, max and mean) and particle morphology (aspect ratio) of the anatase NM-102, rutile NM-103 and anatase/rutile NM-105 were quite comparable to our previous results and to the literature [14], and were not changed after the digestion (Table 3). Other studies have already reported that anatase TiO_2_ (100–200 nm) were biodurable (meaning resistant to chemical/biochemical alteration) in simulated gastric intestinal fluids [26]. The size range of the agglomerates/aggregates as observed by TEM does not change significantly after digestion (*p* > 0.05, Mann–Whitney U test). NM-105 showed the smallest sizes of agglomerates/aggregates by TEM, especially in digested samples, in accordance with the DLS observations.

Surface charge did not show major differences between undigested and digested samples. While the DLS method showed subtle changes in hydrodynamic size of digested NM-105, TEM-EDS supported this view, for this TiO_2_, and further suggested that the other NMs were not clearly modified by the digestion process.

### 3.3. Cytotoxicity 

**Figure 4:**
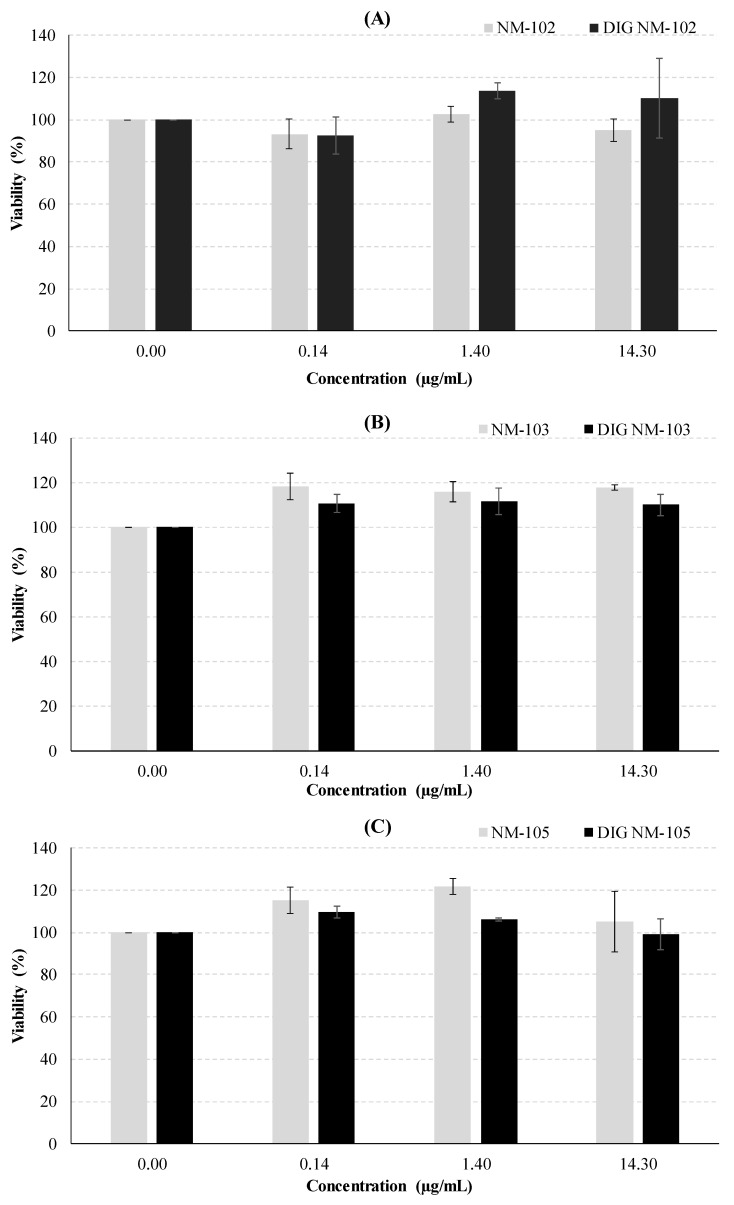
Analysis of the cytotoxic effects in Caco-2 cells, 24 h after exposure to the undigested and digested (DIG) NMs: (**A**) NM-102, (**B**) NM-103 and (**C**) NM-105.

After 24 h of exposure of Caco-2 cells, none of the undigested or digested NMs led to a significant decrease in cell survival (*p* ≥ 0.05 one-way ANOVA test), thus not cytotoxic in the 0.14–14.3 µg/mL concentration range. No clear differences were observed between digested and undigested NMs (Figure 4), except a slight increase in viability seen after exposure of Caco-2 cells to TiO_2_. However, such an increase was only significant in the case of 14.3 µg/mL of NM-103 (*p* = 0.0094, Dunnett’s T3, post-hoc test) and 1.4 µg/mL of DIG NM105 (*p* = 0.034), in comparison to negative controls.

Possible interference of the NMs with the spectrophotometric measurements used in cytotoxicity assays has been reported in the literature [21], where the presence of NMs in the culture media was associated with interference due to the absorption of TiO_2_ NMs at wavelengths used for quantification [21]. In previous studies we tested this kind of interference of TiO_2_ NMs by comparing the absorbance before and after plates centrifugation and transfer of the supernatant to a clean 96-well plate [35]. Given that no differences were detected between the two colorimetric measurements, we assumed no significant interference of the NMs. Furthermore, in studies that report this interference [21], it was observed at much higher concentrations of NM (100 µg/mL) and did not occur with the lower concentrations used in the present study.

The absence of cytotoxicity of the TiO_2_ in Caco-2 cells is in agreement with previous reports [24,25,36,37]. Although Gerloff et al. [36] and Dorier et al. [24,37] did not use exactly the same benchmark NMs, they used TiO_2_ very similar to ours. Gerloff et al. used anatase with 21.5 nm (NM-102 has 22 nm) [36] and Dorier et al. used P25, a mixture of anatase and rutile [37], which is referenced in the NM repository of the Joint Research Centre (JRC, Ispra, Italy) as very similar to NM-105. After 24 h of exposure, both NMs were unable to produce cytotoxic effects in Caco-2 cells using LDH (20 and 80 µg/cm^2^) and WST-1 assays (0–200 µg/mL), respectively [24,37]. More recently, using the MTT assay, it was also verified that, after 24 h of exposure, the anatase-rutile mixture NM-105 (0–200 µg/mL) did not induce cytotoxic effects in a co-culture system of Caco-2 and HT29-MTX intestinal cells [24]. Using the neutral red uptake assay, Jalili et al. reported no cytotoxic effects in Caco-2 differentiated monolayer cells after 24 h of exposure to NM-103 (0–256 µg/mL) [25]. Likewise, no cytotoxic effect was verified in Caco-2 cells exposed to anatase TiO_2_ (NM-100, <100 nm). Our results together with the results available from the literature indicate that undigested TiO_2_ are not cytotoxic to Caco-2 cells.

With regard to the cytotoxicity of digested NMs, assessed through the MTT assay, few authors reported comparable experiments. McCracken et al. observed a slight reduction in viability after 24 h of exposure of C2BBe1 cells (a cell clone of Caco-2) to 10 µg/cm^2^ (approximately 40 µg/mL) of digested anatase-rutile mixture (21 nm) [38]. The digestion model used by these authors displays relevant differences compared to the most recent in vitro digestion process described by in the present work [21], namely, it does not include an oral phase simulation and it uses different enzyme activities, bile salts concentration and incubation times. Besides, between steps, NMs were pelleted by centrifugation [38]. Conversely, the work by Zhang et al. used food grade TiO_2_ (E171) and a different digestion protocol for exposing a tricellular culture model of Caco-2, HT29-MTX-E12 and Raji B cells, where the highest proportion corresponded to Caco-2 cells [17]. They showed that a concentration of 0.75% (*w*/*w*) of TiO_2_ appeared to reduce the digestion product toxicity [17]. Subsequently, this group reported a slight cytotoxic effect of the same food grade TiO_2_ using the fasting food model, but no cytotoxicity in the standardized food model [27], further supporting our observations.

**Figure 5:**
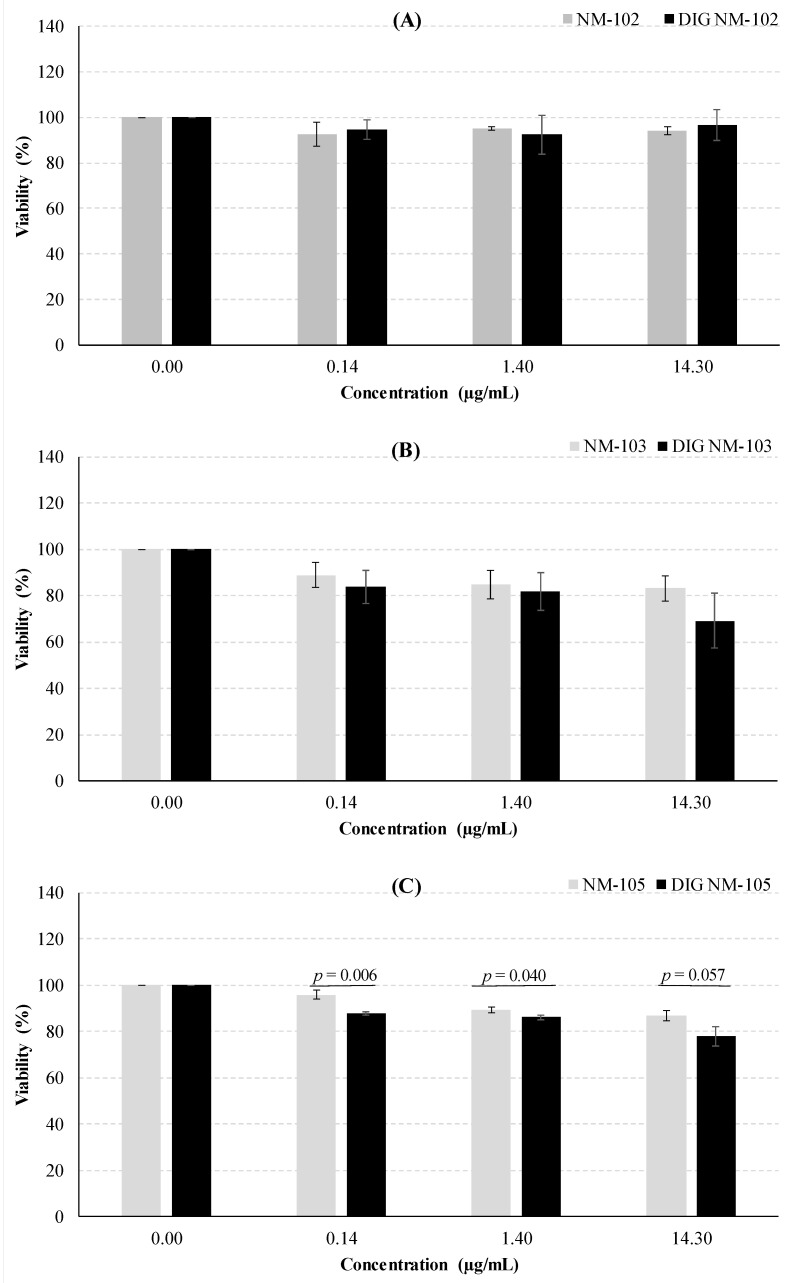
Analysis of the cytotoxic effects in HT29-MTX-E12 cells, 24 h after exposure to the undigested and digested (DIG) NMs: (**A**) NM-102, (**B**) NM-103 and (**C**) NM-105.

In HT29-MTX-E12 cells, while no significant effect was observed after NM-102 exposure (Figure 5), there were significant decreases in the viability after NM-103 exposure, both for the undigested and digested NM (*p* = 0.0338 and *p* = 0.0330, respectively; one-way ANOVA test). Likewise, both undigested and digested NM-105 induced significant decreases in cells’ viability (*p* = 0.0001 and *p* = 0.00007, respectively, one-way ANOVA). The concentrations of 1.40 and 14.3 µg/mL showed significant decreases in viability for undigested NM-105, and the concentrations of 0.14 and 1.40 µg/mL of digested NM-105 significantly decreased the viability (*p* < 0.05, Dunnett’s T3 post-hoc tests). In addition, undigested NM-105 effects fitted a quadratic function (*R^2^* = 0.8669, *p* = 0.00010 ANOVA test), showing a dose-response effect.

In the literature, there are few reports on the cytotoxic effects of TiO_2_ in HT29-MTX-E12 cells. As a matter of fact, only studies related to cytotoxicity and cell viability assays in HT29 cells (which do not have a mucus-secreting phenotype) exposed to TiO_2_ have been found. Ammendolia et al. used anatase TiO_2_, with a primary size < 25 nm, similar to NM-102 but with a different surface area (45–55 m^2^/g compared to NM-102 with 77.99 m^2^/g) [39]. Using the MTT and LDH assays, it was possible to infer that after 6, 24 and 48 h of exposure of HT29 cells to TiO_2_ (1–20 μg/cm^2^) there were no significant cytotoxic effects [40]. In another report, HT29 cells were exposed for 48 h to higher concentrations of TiO_2_ (50, 100, 200 and 400 μg/mL) [40] and a 20–30% decrease in cell viability occurred, detected by the MTT assay, only at the lowest and highest concentrations. The authors concluded that cell viability was affected by the TiO_2_ in a dose-dependent way [40]. In our report, despite a different exposure duration (24 h) and a much lower concentration range of TiO_2_, it was also verified that the increase in NM-103 and NM-105 concentrations promoted a decrease in cell viability and, therefore, an increase in cytotoxicity in HT29-MTX-E12 cells. Using the trypan blue exclusion test and the MTT assay, Schneider et al. [41], also reported a significant decrease of the membrane integrity after trypan blue staining, although they did not observe effects on cell viability by MTT on HT29 cells after 24 h treatment with 2–10 μg/mL of TiO_2_ similar to NM-105 (anatase/rutile, particle size 27.38 ± 5.90 nm,). However, it must be noted that the results from other studies presented above may differ from our own results, since the cell lines HT29 and HT29-MTX-E12 have different characteristics. The HT29-MTX-E12 cell line consists of a homogeneous population of gastric mucin-producing cells, unlike the HT29 cell line, which has about 95% of undifferentiated cells and a reduced proportion of mucus-secreting cells (i.e., <5%) [42,43]. No reports were found regarding the viability of HT29-MTX-E12 cells after exposure to digested TiO_2_.

In the present study, the comparison of the undigested versus digested NM did not reveal significant differences, except for NM-105 in HT29-MTX-E12 cells, where, at each concentration, the digested NM decreased significantly the viability (*p* ≤ 0.05, Student’s *t*-test). A similar pattern was observed following digestion of NM-103, but the results were not significant (Figure 5). The significantly lower size of agglomerates/aggregates shown by DLS in digested NM-105 as compared to undigested NM may be responsible for the differences in cellular toxicity. Previously, we have shown that there was a significant correlation between the hydrodynamic size of TiO_2_ in the cell medium and the level of DNA damage [13,15], where increased sizes yielded lower DNA damage. Smaller NMs may be more easily uptaken by cells, thus exerting a potentially higher adverse impact on cell functioning that could explain higher toxicity. A mechanism of reactive oxygen species (ROS) induction has been frequently related to nanotoxicity [44]. In fact, a recent work showed an intracellular increase of reactive oxygen species (ROS) upon exposure of normal human colon cell lines (NCM460) and human colon cancer cells (HCT116) to TiO_2_, that was suggested as the cause of its cytotoxicity and was related to microRNAs 378b and 378 g expression [45]. Increased uptake of smaller TiO_2_ might favor this type of mechanisms, explaining the results observed in HT29-MTX-E12 cells exposed to digested NM-105, and deserves further research. However, it cannot be discarded that other properties, such as surface area or dissolution behavior, may have been influenced by the digestion process.

The two intestinal cell lines used in this work (i.e., Caco-2 and HT29-MTX-E12) presented different levels of sensitivity to NMs exposure. The Caco-2 cell line showed no significant differences in cell viability when exposed to either digested or undigested TiO_2_ nanomaterials. An explanatory hypothesis for this might be the fact that the used cells have a non-differentiated form and, therefore, do not express certain biochemical and morphological features, which are characteristic of human absorptive enterocytes [24]. However, in different reports it has been described that undifferentiated Caco-2 cells have a high sensitivity to nanomaterials, namely TiO_2_ [46], SiO_2_ and ZnO [47], meaning that a smaller dose of particles is needed to obtain greater toxicity in the cells. This conclusion was not observed in our work, so the contradictory results that are observed in vitro might be caused by different culture conditions, the use of NMs with different physical-chemical properties (i.e., size) [46] and/or a distinct preparation procedure of the NMs [24]. Since the agglomeration/aggregation behavior seems to be determinant to the biological outcome, the use of different cell culture media may justify the discrepancies in the sensitivity reported in the literature.

In contrast, HT29-MTX-E12 cells showed a significant decrease in viability when exposed to NM-105 (either in the digested or undigested form). It was also observed that the level of cytotoxicity is much higher when compared to the cell line Caco-2, so these cells may have a higher sensitivity. As previously mentioned, HT29-MTX-E12 cells secrete mucus, which can cover them and consequently protect the cells from NMs, thus promoting a possible lower internalization [24]. This observation is not in agreement with the results obtained, which might be due to the fact that cells not having enough time in culture to start producing high amounts of mucus in such a way that it is possible to fully cover the cells, thus protecting them. Dorier et al. (2019b) reported that HT29-MTX cells already produced some mucus, in three days post-seeding. Additionally, in a co-culture of Caco-2/HT29-MTX exposed to TiO_2_ (95% anatase, 12 ± 3 nm), the NMs were mainly accumulated in the HT29-MTX cells [48]. The authors explained this event by the fact that Goblet cells (HT29-MTX-E12 cells are derived from them) may undergo an endocytosis process, although in mature enterocytes it is not possible [48,49]. Thus, different endocytic capacity of Caco-2 and HT29-MTX-E12 cells may explain the different sensitivity to the cytotoxicity induced by NM-105, especially after digestion simulation, when the agglomerates/aggregates size is smaller.

This work shows the challenges when addressing the NMs toxicity, since data interpretation and, therefore, hazard assessment may be influenced by the secondary properties underlying the test system or by the cell model selected that should mimic accurately the nano-cellular interactions in the target organ.

## 4. Conclusions

In this study, we characterized the NMs transformations and assessed their cytotoxicity after the digestion process. Overall, we show that the physicochemical properties should be accounted for when interpreting the toxicological data, in conditions mimicking the in vivo environment [50].

In spite of the technical challenges due to the complexity of the digestion medium, the standardized INFOGEST 2.0 in vitro digestion method in combination with DLS, zeta potential and TEM-EDS methodologies provided insights into the factors influencing the cytotoxicity data.

In one of the intestinal cell lines exposed to NM-105, a more pronounced adverse outcome was shown after exposure to the digestion product, as compared to undigested NM, concomitantly with subtle changes in characteristics of the NM, suggesting the importance to include the simulation of digestion to improve in vitro studies related to gastrointestinal tract toxicity of nanomaterials. In can be considered that many of the (contradictory) results in the literature cited are underestimating the hazard due to the lack of consideration of the human digestion process in NMs’ toxicity. Future experiments may use this digestion simulation for improved nanosafety assessment in non-cancer intestinal cells or in more complex co-culture models. As perceivable from the literature, fundamental studies focusing on specific endpoints and cell lines, evaluating a small number of variables are an essential first step to build a consistent body of data, and suggesting successful or unsuccessful approaches in the hard process of NMs nanotoxicological evaluation. It is foreseen that this model can be a valuable tool for in depth addressing ingested NMs’ toxicity and genotoxicity in future in vitro studies, thereby reducing uncertainties in the risk assessment of ingested NMs for human health.

## Figures and Tables

**Table 1:** The physicochemical characteristics of the titanium dioxide nanomaterials [28].

	NM-102	NM-103	NM-105
Batch#	JRCNM10202a	JRCNM62001a	JRCNM01005a
Crystalline phase	Anatase	Rutile	Anatase and rutile (81.5:18.5.)
Primary particle size (nm)	22	28	30
DLS (nm)	Zav = 442.6 PDI = 0.428	Zav = 113.8 PDI = 0.252	Zav = 124.5 PDI = 0.172
Specific surface area (m^2^/g)	77.99	50.84	46.18
Agglomerates/ aggregates size (nm)	100–500	NA	20–500

DLS, dynamic light scattering; Zav, hydrodynamic particle size; PDI, polydispersity index; NA, not available. For DLS measurements, suspensions of TiO_2_ nanomaterials were dispersed by ultrasonication in BSA water according to the validated NANOGENOTOX protocol [29].

**Table 2:** DLS measurements of hydrodynamic mean size (Zav, nm), polydispersity index (PDI) and surface charge (Zeta potential, mV) of the nanomaterials (NMs) dispersions in cell culture medium (14 µg/mL), with and without digestion simulation. Results are presented as mean ± SD (*n* = 3).

NM	Undigested			Digested		
	Zav (nm)	PDI	Zeta Potential (mV)	Zav (nm)	PDI	Zeta Potential (mV)
NM-102	551.30 ± 46.53	0.49 ± 0.14	−22.0 ± 2.8	560.78 ± 254.75	0.70 ± 0.08	−21.4 ± 1.5
NM-103	545.10 ± 490.87	0.58 ± 0.43	−27.0 ± 0.6	478.60 ± 199.97	0.77 ± 0.15	−26.8 ± 0.4
NM-105	292.40 ± 0.85	0.73 ± 0.21	−22.2 ± 2.0	190.40 * ± 9.33	0.52 ± 0.36	−26.7 ± 0.8

* significantly different from undigested sample (*p* = 0.0042, Student’s *t*-test).

**Table 3:** Quantitative analysis of size and aspect ratio of the primary particles (geometric mean ± geometric standard deviation) and size range of the agglomerates/aggregates in the samples.

		Feret Min (nm)±	Feret Max (nm)*	Feret Mean (nm)	% of Particles < 100 nm	Aspect Ratio	Agglomerate/ Aggregate Size Range (nm)
**NM-102**	U	20.2 ± 1.1	29.8 ± 1.3	24.6 ± 1.3	100	1.5 ± 1.3	111.8–315.0
	D	21.2 ± 1.1	31.2 ± 1.1	25.7 ± 1.1	100	1.5 ± 1.1	123.9–334.0
**NM-103**	U	16.6 ± 1.2	30.1 ± 1.4	22.4 ± 1.4	100	1.8 ± 1.4	102.3–400.4
	D	16.7 ± 1.3	29.6 ± 1.4	22.2 ± 1.4	100	1.8 ± 1.4	94.3–198.2
**NM-105**	U	17.6 ± 1.3	24.3 ± 1.2	20.7 ± 1.2	100	1.4 ± 1.2	66.4–162.2
	D	18.3 ± 1.3	25.4 ± 1.3	21.6 ± 1.3	100	1.4 ± 1.3	48.7–195.5

U, undigested sample; D, digested sample.

## References

[1] Peters R., Weigel S., Marvin H., Bouwmeester H., Aschberger K., Rauscher H., Amenta V., Arena M., Botelho Moniz F., Gottardo S. (2014). Inventory of Nanotechnology Applications in the Agricultural, Feed and Food Sector.

[2] Kumar S., Bhanjana G., Sharma A., Dilbaghi N., Sidhu M.C., Kim K.-H. (2017). Development of nanoformulation approaches for the control of weeds. Sci. Total Environ..

[3] Gómez H.C., Serpa A., Velásquez-Cock J., Gañán P., Castro C., Vélez L., Zuluaga R. (2016). Vegetable nanocellulose in food science: A review. Food Hydrocoll..

[4] Mercier-Bonin M., Despax B., Raynaud P., Houdeau E., Thomas M. (2018). Mucus and microbiota as emerging players in gut nanotoxicology: The example of dietary silver and titanium dioxide nanoparticles. Crit. Rev. Food Sci. Nutr..

[5] Shi H., Magaye R., Castranova V., Zhao J. (2013). Titanium dioxide nanoparticles: A review of current toxicological data. Part. Fibre Toxicol..

[6] Bettini S., Boutet-Robinet E., Cartier C., Coméra C., Gaultier E., Dupuy J., Naud N., Taché S., Grysan P., Reguer S. (2017). Food-grade TiO_2_ impairs intestinal and systemic immune homeostasis, initiates preneoplastic lesions and promotes aberrant crypt development in the rat colon. Sci. Rep..

[7] Pele L.C., Thoree V., Bruggraber S.F.A., Koller D., Thompson R.P.H., Lomer M.C., Powell J.J. (2015). Pharmaceutical/food grade titanium dioxide particles are absorbed into the bloodstream of human volunteers. Part. Fibre Toxicol..

[8] Böckmann J., Lahl H., Eckert T., Unterhalt B. (2000). Titan-Blutspiegel vor und nach Belastungsversuchen mit Titandioxid\r[Blood titanium levels before and after oral administration titanium dioxide]\r. Pharmazie.

[9] Heringa M.B., Peters R.J.B., Bleys R.L.A.W., Van der Lee M.K., Tromp P.C., Van Kesteren P.C.E., Van Eijkeren J.C.H., Undas A.K., Oomen A.G., Bouwmeester H. (2018). Detection of titanium particles in human liver and spleen and possible health implications. Part. Fibre Toxicol..

[10] EFSA (2016). Re-evaluation of titanium dioxide (E 171) as a food additive. EFSA J..

[11] EFSA (2019). EFSA statement on the review of the risks related to the exposure to the food additive titanium dioxide (E 171) performed by the French Agency for Food, Environmental and Occupational Health and Safety (ANSES). EFSA J..

[12] Anses (2019). AVIS de l’Agence Nationale de Sécurité Sanitaire de L’alimentation, de L’environnement et du Travail Relatif Aux Risques Liés à L’ingestion de L’additif Alimentaire E171.

[13] Louro H., Saruga A., Santos J., Pinhão M., Silva M.J. (2019). Biological impact of metal nanomaterials in relation to their physicochemical characteristics. Toxicol. Vitr..

[14] Tavares A.M., Louro H., Antunes S., Quarré S., Simar S., De Temmerman P.J., Verleysen E., Mast J., Jensen K.A., Norppa H. (2014). Genotoxicity evaluation of nanosized titanium dioxide, synthetic amorphous silica and multi-walled carbon nanotubes in human lymphocytes. Toxicol. Vitr..

[15] Louro H. (2018). Relevance of physicochemical characterization of nanomaterials for understanding nano-cellular interactions. Adv. Exp. Med. Biol..

[16] Sohal I.S., O’Fallon K.S., Gaines P., Demokritou P., Bello D. (2018). Ingested engineered nanomaterials: State of science in nanotoxicity testing and future research needs. Part. Fibre Toxicol..

[17] Zhang Z., Zhang R., Xiao H., Bhattacharya K., Bitounis D., Demokritou P., McClements D.J. (2019). Development of a standardized food model for studying the impact of food matrix effects on the gastrointestinal fate and toxicity of ingested nanomaterials. NanoImpact.

[18] DeLoid G.M., Wang Y., Kapronezai K., Lorente L.R., Zhang R., Pyrgiotakis G., Konduru N.V., Ericsson M., White J.C., De La Torre-Roche R. (2017). An integrated methodology for assessing the impact of food matrix and gastrointestinal effects on the biokinetics and cellular toxicity of ingested engineered nanomaterials. Part. Fibre Toxicol..

[19] Minekus M., Alminger M., Alvito P., Ballance S., Bohn T., Bourlieu C., Carrière F., Boutrou R., Corredig M., Dupont D. (2014). A standardised static in vitro digestion method suitable for food-an international consensus. Food Funct..

[20] Egger L., Ménard O., Delgado-Andrade C., Alvito P., Assunção R., Balance S., Barberá R., Brodkorb A., Cattenoz T., Clemente A. (2016). The harmonized INFOGEST in vitro digestion method: From knowledge to action. Food Res. Int..

[21] Brodkorb A., Egger L., Alminger M., Alvito P., Assunção R., Ballance S., Bohn T., Bourlieu-Lacanal C., Boutrou R., Carrière F. (2019). INFOGEST static in vitro simulation of gastrointestinal food digestion. Nat. Protoc..

[22] Improving Health Properties of Food by Sharing our Knowledge on the Digestive Process - Home Page. https://www.cost-infogest.eu/.

[23] Tan Y., Li R., Zhou H., Liu J., Muriel Mundo J., Zhang R., McClements D.J. (2020). Impact of calcium levels on lipid digestion and nutraceutical bioaccessibility in nanoemulsion delivery systems studied using standardized INFOGEST digestion protocol. Food Funct..

[24] Dorier M., Tisseyre C., Dussert F., Béal D., Arnal M.E., Douki T., Valdiglesias V., Laffon B., Fraga S., Brandão F. (2019). Toxicological impact of acute exposure to E171 food additive and TiO_2_ nanoparticles on a co-culture of Caco-2 and HT29-MTX intestinal cells. Mutat. Res. Genet. Toxicol. Environ. Mutagen..

[25] Jalili P., Gueniche N., Lanceleur R., Burel A., Lavault M.-T., Sieg H., Böhmert L., Meyer T., Krause B.-C., Lampen A. (2018). Investigation of the *In Vitro* genotoxicity of two rutile TiO_2_ nanomaterials in human intestinal and hepatic cells and evaluation of their interference with toxicity assays. NanoImpact.

[26] Sohal I.S., Cho Y.K., O’Fallon K.S., Gaines P., Demokritou P., Bello D. (2018). Dissolution Behavior and Biodurability of Ingested Engineered Nanomaterials in the Gastrointestinal Environment. ACS Nano.

[27] Cao X., Zhang T., De Loid G.M., Gaffrey M.J., Weitz K.K., Thrall B.D., Qian W.J., Demokritou P. (2020). Evaluation of the cytotoxic and cellular proteome impacts of food-grade TiO_2_ (E171) using simulated gastrointestinal digestions and a tri-culture small intestinal epithelial model. NanoImpact.

[28] Rasmussen K., Mast J., De Temmerman P., Verleysen E., Waegeneers N., Van Steen F., Pizzolon J.C., De Temmerman L., Jensen K.A., Birkedal R. (2014). Titanium Dioxide, NM-100, NM-101, NM-102, NM-103, NM-104, NM-105: Characterisation and Physico—Chemical Properties. Science and Policy Report by the Joint Research Centre of the European Commission.

[29] Jensen K.A., Kembouche Y., Christiansen E., Jacobsen N.R., Wallin H., Guiot C., Spalla O., Witschger O. (2011). Final Protocol for Producing Suitable Manufactured Nanomaterial Exposure Media. anses.fr/en/system/files/nanogenotox_deliverable_5.pdf.

[30] Richter J.W., Shull G.M., Fountain J.H., Guo Z., Musselman L.P., Fiumera A.C., Mahler G.J. (2018). Titanium dioxide nanoparticle exposure alters metabolic homeostasis in a cell culture model of the intestinal epithelium and Drosophila melanogaster. Nanotoxicology.

[31] Guo Z., Martucci N.J., Moreno-Olivas F., Tako E., Mahler G.J. (2017). Titanium dioxide nanoparticle ingestion alters nutrient absorption in an in vitro model of the small intestine. NanoImpact.

[32] DeLoid G.M., Cohen J.M., Pyrgiotakis G., Demokritou P. (2017). An integrated dispersion preparation, characterization and in vitro dosimetry methodology for engineered nanomaterials. Nat. Protoc..

[33] Bhattacharjee S. (2016). DLS and zeta potential - What they are and what they are not?. J. Control. Release.

[34] Li Q., Li T., Liu C., De Loid G., Pyrgiotakis G., Demokritou P., Zhang R., Xiao H., McClements D.J. (2017). Potential impact of inorganic nanoparticles on macronutrient digestion: Titanium dioxide nanoparticles slightly reduce lipid digestion under simulated gastrointestinal conditions. Nanotoxicology.

[35] Saruga A.S.F. (2016). Cyto-and Genotoxicity Assessment of Manufactured Nanomaterials in the A549 Cell Line. Master’s Thesis.

[36] Gerloff K., Fenoglio I., Carella E., Kolling J., Albrecht C., Boots A.W., Förster I., Schins R.P.F. (2012). Distinctive toxicity of TiO_2_ rutile/anatase mixed phase nanoparticles on Caco-2 cells. Chem. Res. Toxicol..

[37] Dorier M., Béal D., Marie-Desvergne C., Dubosson M., Barreau F., Houdeau E., Herlin-Boime N., Carriere M. (2017). Continuous in vitro exposure of intestinal epithelial cells to E171 food additive causes oxidative stress, inducing oxidation of DNA bases but no endoplasmic reticulum stress. Nanotoxicology.

[38] McCracken C., Zane A., Knight D.A., Dutta P.K., Waldman W.J. (2013). Minimal intestinal epithelial cell toxicity in response to short- and long-term food-relevant inorganic nanoparticle exposure. Chem. Res. Toxicol..

[39] Ammendolia M.G., Iosi F., Maranghi F., Tassinari R., Cubadda F., Aureli F., Raggi A., Superti F., Mantovani A., De Berardis B. (2017). Short-term oral exposure to low doses of nano-sized TiO_2_ and potential modulatory effects on intestinal cells. Food Chem. Toxicol..

[40] Kukia N.R., Rasmi Y., Abbasi A., Koshoridze N., Shirpoor A., Burjanadze G., Saboory E. (2018). Bio-effects of TiO_2_ nanoparticles on human colorectal cancer and umbilical vein endothelial cell lines. Asian Pac. J. Cancer Prev..

[41] Schneider T., Westermann M., Glei M. (2017). In Vitro uptake and toxicity studies of metal nanoparticles and metal oxide nanoparticles in human HT29 cells. Arch. Toxicol..

[42] Huet G., Kim I., De Bolos C., Lo-Guidice J.M., Moreau O., Hemon B., Richet C., Delannoy P., Real F.X., Degand P. (1995). Characterization of mucins and proteoglycans synthesized by a mucin-secreting HT-29 cell subpopulation. J. Cell Sci..

[43] Gagnon M., Zihler Berner A., Chervet N., Chassard C., Lacroix C. (2013). Comparison of the Caco-2, HT-29 and the mucus-secreting HT29-MTX intestinal cell models to investigate Salmonella adhesion and invasion. J. Microbiol. Methods.

[44] Song B., Zhou T., Yang W.L., Liu J., Shao L.Q. (2016). Contribution of oxidative stress to TiO_2_ nanoparticle-induced toxicity. Environ. Toxicol. Pharmacol..

[45] Li W., Jia M.X., Deng J., Wang J.H., Zuberi Z., Yang S., Ba J., Chen Z. (2020). MicroRNA response and toxicity of potential pathways in human colon cancer cells exposed to titanium dioxide nanoparticles. Cancers.

[46] Song Z.M., Chen N., Liu J.H., Tang H., Deng X., Xi W.S., Han K., Cao A., Liu Y., Wang H. (2015). Biological effect of food additive titanium dioxide nanoparticles on intestine: An In Vitro study. J. Appl. Toxicol..

[47] Gerloff K., Pereira D.I.A., Faria N., Boots A.W., Kolling J., Förster I., Albrecht C., Powell J.J., Schins R.P.F. (2013). Influence of simulated gastrointestinal conditions on particle-induced cytotoxicity and interleukin-8 regulation in differentiated and undifferentiated Caco-2 cells. Nanotoxicology.

[48] Brun E., Barreau F., Veronesi G., Fayard B., Sorieul S., Chanéac C., Carapito C., Rabilloud T., Mabondzo A., Herlin-Boime N. (2014). Titanium dioxide nanoparticle impact and translocation through ex vivo, in vivo and in vitro gut epithelia. Part. Fibre Toxicol..

[49] Hansen G.H., Rasmussen K., Niels-Christiansen L.L., Danielsen E.M. (2009). Endocytic trafficking from the small intestinal brush border probed with FM dye. Am. J. Physiol. Gastrointest. Liver Physiol..

[50] Graça D., Louro H., Santos J., Dias K., Almeida A.J., Gonçalves L., Silva M.J., Bettencourt A. (2017). Toxicity screening of a novel poly(methylmethacrylate)-Eudragit nanocarrier on L929 fibroblasts. Toxicol. Lett..

